# A fully recyclable heterogenized Cu catalyst for the general carbene transfer reaction in batch and flow†
†Electronic supplementary information (ESI) available: Experimental details of catalyst preparation, catalytic experiments and identification of the products. See DOI: 10.1039/c4sc03277b



**DOI:** 10.1039/c4sc03277b

**Published:** 2014-11-28

**Authors:** Lourdes Maestre, Erhan Ozkal, Carles Ayats, Álvaro Beltrán, M. Mar Díaz-Requejo, Pedro J. Pérez, Miquel A. Pericàs

**Affiliations:** a Laboratorio de Catálisis Homogénea , Unidad Asociada al CSIC , CIQSO-Centro de Investigación en Química Sostenible and Departamento de Química y Ciencia de Materiales , Universidad de Huelva , 21007 Huelva , Spain . Email: perez@dqcm.uhu.es ; Email: mmdiaz@dqcm.uhu.es ; Tel: +34 959 219956; b Institute of Chemical Research of Catalonia (ICIQ) , Avda. Països Catalans 16 , 43007 , Tarragona , Catalonia , Spain . Email: mapericas@iciq.es ; Tel: +34 977 920 243; c Departament de Química Orgànica , Universitat de Barcelona , 08028 Barcelona , Spain

## Abstract

A polystyrene-linked tris(triazolyl)methanecopper(i) cationic catalyst operates under heterogeneous conditions for the reaction of ethyl diazoacetate (EDA) with an array of substrates. Carbon–hydrogen as well as X–H (X = O, N) functionalization derived from the formal transfer of the carbene moiety (:CHCO_2_Et) from the copper center and subsequent insertion have been achieved, the reactions permitting repeated catalyst recycling and reuse. The addition of the same carbene unit to benzene leading to a cycloheptatriene derivative (Büchner reaction) or to phenylacetylene (cyclopropenation) took place at similar rates to the insertion processes and with the same catalyst recyclability. The use of this heterogenized cationic Cu catalyst in continuous flow has also been implemented. Key characteristics of the flow process are its high and constant turnover frequency (TOF) (residence times of 1 min still lead to full conversion in the reaction with ethanol after 48 h operation) and its suitability for the sequential performance of different types of carbene transfer reactions with a simple and affordable experimental setup.

## Abstract 2

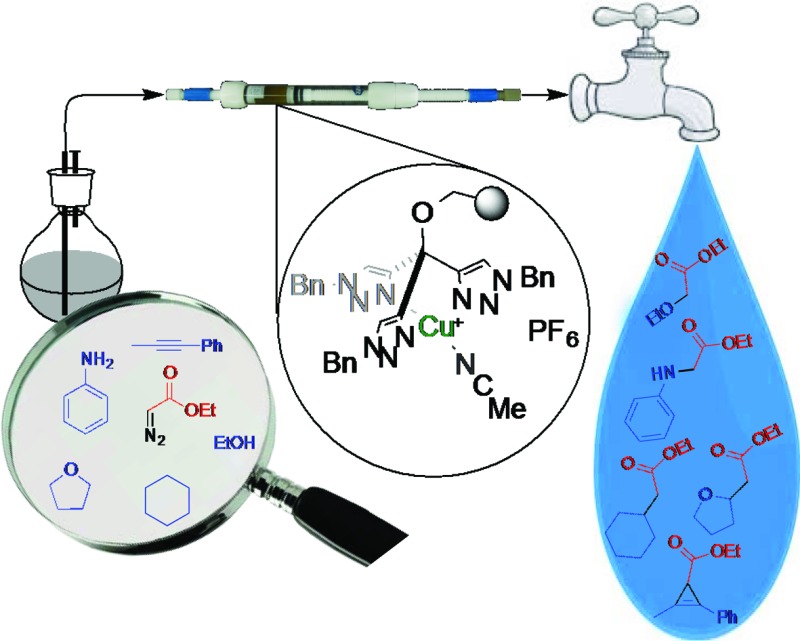
Carbene transfer reactions can be performed in batch and flow with a highly active, chemically stable heterogenized tris(triazolyl)methyl copper(i) catalyst.

## Introduction

**Scheme 1:**
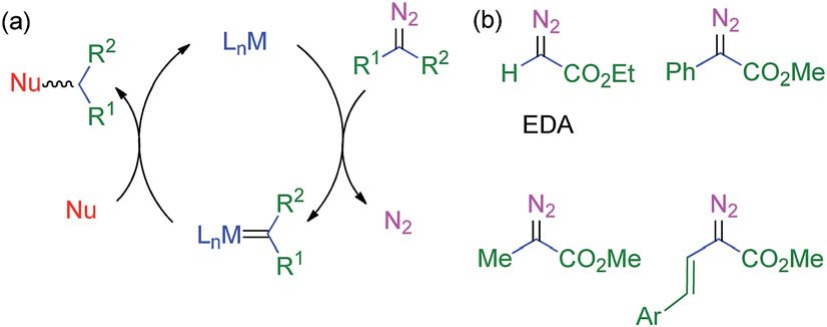
(a) the metal-catalyzed transfer of carbene groups from diazo compounds. (b) commonly employed diazo reagents.

**Scheme 2:**
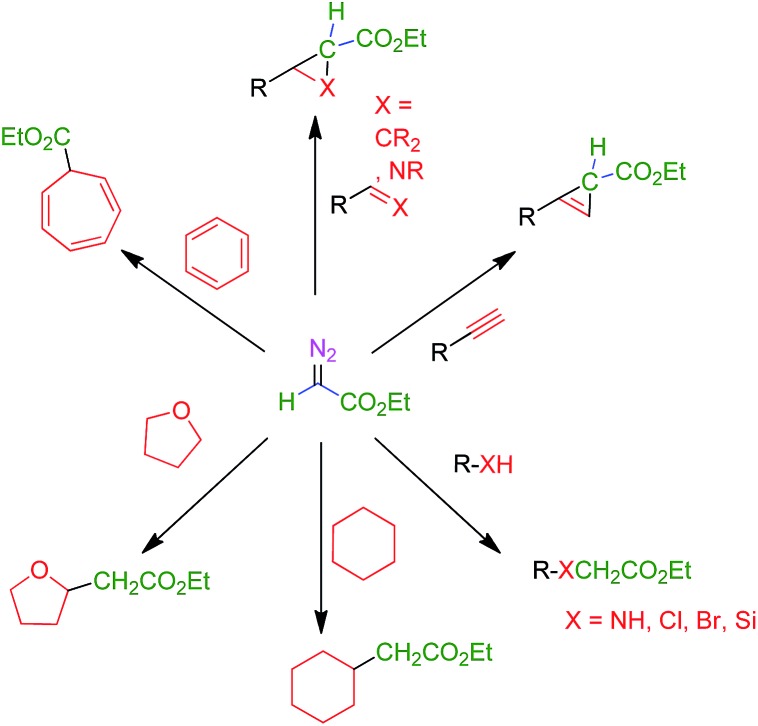
Functionalization of organic substrates by metal-catalyzed carbene transfer addition or insertion reactions.

The use of transition metal-based catalysts for *in situ* diazo compound decomposition and carbene transfer to saturated or unsaturated substrates constitutes a widely employed tool in applied organometallic chemistry.^1,2^ This reaction occurs through a metallocarbene intermediate, from which the carbene ligand is transferred to a nucleophile, leading to the net functionalization of the latter (Scheme 1a). This strategy has been applied to a large number of substrates such as alkenes, alkynes, imines and arenes as unsaturated reactants, and several X–H bonds (X = C, O, N, Si) and C–halogen bonds, in saturated compounds. Well-established protocols for highly efficient asymmetric transformations are also known.^1^ Ethyl diazoacetate (EDA) is widely employed both at the laboratory and industrial scale (*e.g.* the Sumitomo process for olefin cyclopropanation), with other substituted diazo compounds being increasingly employed (Scheme 1b), mainly due to the beneficial effect of the presence of both acceptor and donor substituents at the carbon bearing the diazo functionality. Scheme 2 shows examples of the different transformations reported to date with ethyl diazoacetate as the carbene source and with transition metal complexes as catalysts.

The aforementioned transformations (Scheme 2) have been mainly developed with soluble catalysts, under homogeneous conditions. Therefore, they suffer from the expected difficulties associated with catalyst separation that, in most cases, lead to zero recovery of the catalyst. Heterogeneous catalysts for carbene transfer reactions are scarce. Processes studied with these catalysts have been limited to olefin cyclopropanation,^3^ a few examples of carbene insertion into C–H bonds^4^ or N–H bonds,^5^ and alkyne cyclopropenation.^6^ We therefore considered that the development of a catalytic system capable of operating under heterogeneous conditions and, more importantly, being active enough to promote all the reactions shown in Scheme 2 would represent a significant advance. The target catalyst would ideally exhibit high chemical and mechanical stability, thus allowing repeated recycling. As an ultimate goal, the implementation of efficient flow versions of the reactions being considered was sought.

Flow processing^7^ based on heterogenized catalysts in packed bed reactors is gaining increasing acceptance for its inherent advantages over conventional batch processing.^8^ Some of the common drawbacks of heterogeneous catalysis in batch, such as mechanical degradation of the catalyst particles originating from continuous stirring and inter-cycle deactivation due to the oxidation and/or hydrolysis of labile catalytic species can be efficiently avoided with the implementation of continuous flow processes.^9^

In spite of its potential, the possibility of performing metal-catalyzed carbene transfer reactions from diazo compounds in continuous flow has received little attention, and only olefin cyclopropanation^10^ and intramolecular C–H insertion^11*a*^ and N–H insertion^11*b*^ reactions have been reported under these conditions.

As the result of a joint effort combining our previous and separate experiences in the areas of carbene transfer, catalyst immobilization and flow chemistry, we wish to report in this contribution the development of a highly efficient heterogenized catalyst for carbene transfer reactions that operates with an almost complete absence of Cu leaching, and that can be repeatedly recycled and reused under batch conditions and used in continuous flow conditions as well for prolonged periods of time.

## Results and discussion

### Preparation of the heterogenized catalyst

**Scheme 3:**
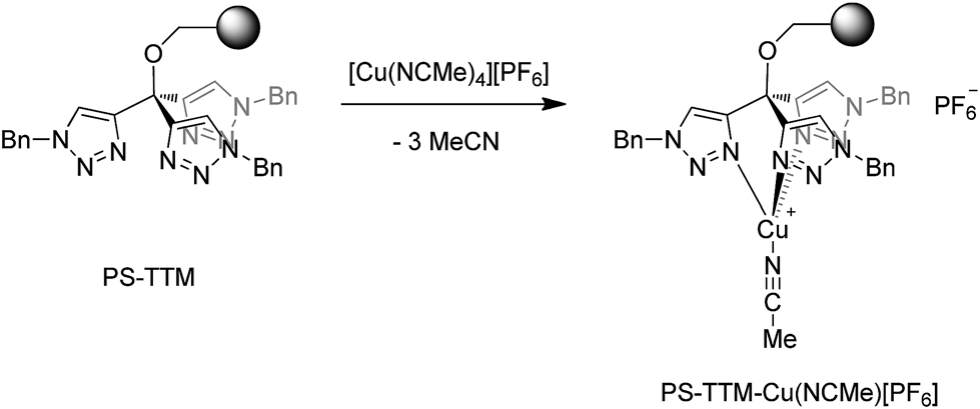
The PS–TTM ligand and its cationic Cu(NCMe) complex.

Very recently the synthesis of a family of tris(triazolyl)methane (TTM) derivatives has been reported, and the corresponding (TTM)CuCl complexes have found application as efficient mediators in the copper-catalyzed alkyne–azide cycloaddition (CuAAC) reaction in a variety of reaction conditions.^12^ Polystyrene-supported versions of the TTM ligands (PS–TTM) have also been developed and, in particular, it has been found that Merrifield resins modified by etherification with TTM units (PS–TTM, Scheme 3) behave as ligands with unlimited recyclablity for CuAAC reactions.^13^ In practice, however, the resin has to be recharged every 5–6 cycles with CuCl due to leaching. We reasoned that a cationic Cu complex would result in a much stronger interaction with the PS–TTM ligand, and that metal leaching could be minimized in this manner. Given the well-known capabilities of copper complexes bearing tripodal ligands with pyrazolyl groups,^14^ we anticipated that the corresponding PS–TTM complexes would exhibit similar catalytic capabilities toward carbene transfer reactions from ethyl diazoacetate.

The reaction of the polystyrene-supported tris(triazolyl)methyl complex (PS–TTM, *f* = 0.495 mmol g^–1^) with [Cu(MeCN)_4_][PF_6_] in dichloromethane at room temperature led to the direct formation of the cationic complex [(PS–TTM)Cu(NCMe)][PF_6_] bearing a labile acetonitrile ligand (Scheme 3). According to copper elemental analysis, the functionalization of the Cu-charged resin is *f* = 0.39 mmol g^–1^, corresponding to a functionalization yield (100*f*/*f*_max_) of 89% (see ESI†).

### Reaction of ethyl diazoacetate and organic substrates with PS–TTM–Cu(NCMe)[PF_6_] as the catalyst: batch conditions

**Scheme 4:**
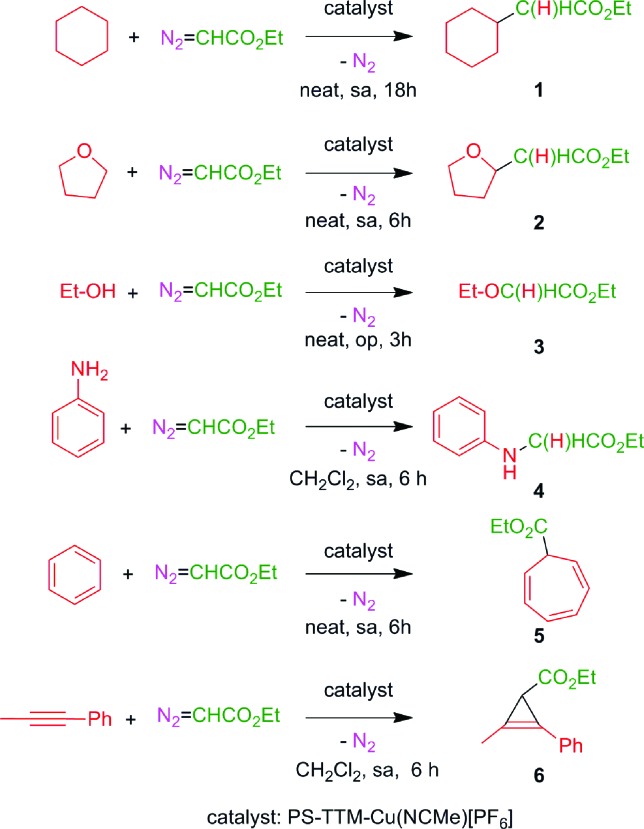
Reactions studied in this work. sa: slow addition of EDA; op: EDA added in one portion.

We have chosen some representative reactions (Scheme 4) to study the capability of [(PS–TTM)Cu(NCMe)][PF_6_] to transfer the carbene group, :CHCO_2_Et, from N_2_C(H)CO_2_Et in a catalytic manner and to evaluate the possibility of catalyst recycling and reuse under batch conditions. The substrates employed were cyclohexane, tetrahydrofuran, ethanol and aniline to assess the catalyst efficiency in insertion reactions, and benzene and 1-phenyl-1-propyne as models for addition reactions (Scheme 4). We have not studied the olefin cyclopropanation reaction given the high number of examples of this process already described under heterogeneous conditions.^8a–c,10^

The general procedure consisted of the preparation of a mixture containing the solid catalyst (5.2 mol%) and the substrate (in excess, neat or dissolved in dichloromethane) and the addition of ethyl diazoacetate (the limiting reagent, in one portion or by slow addition, depending on the substrate) under an inert atmosphere. After complete consumption of EDA, the liquid phase was separated by filtration, the catalyst was washed with dichloromethane (2 × 5 mL), and fresh reactants were added for the next reaction cycle. The results of five consecutive reaction cycles for the six different reactions in Scheme 4, keeping the reaction time constant, are shown in Table 1. In all cases, complete conversion of the starting EDA was recorded. The reaction crudes were in all cases very clean, the only by-products being diethyl fumarate and diethyl maleate arising from the well-known catalytic coupling of two molecules of EDA.

**Table 1:** Recycling of [(PS–TTM)Cu(NCMe)][PF_6_] in the carbene transfer reaction from EDA leading to **1–6** ^*a*^

Products	Catalytic cycle #					Avg.
	1	2	3	4	5	
	Yield^*b*^ (%)					
**1**	98	75	72	71	67	76
**2**	82	86	86	94	95	89
**3**	99^*c*^	99	99	99	99	99
**4**	97^*d*^	99	97	90	86	94
**5**	88	82	80	82	82	83
**6**	93	91	91	89	94	92

^*a*^All reactions were performed with 5.2 mol% of catalyst loading.

^*b*^Yields were determined by GC and NMR.

^*c*^Starting from 4.5 mmol EDA, **3** was obtained with 99% GC yield and 98% isolated yield. See ESI for details.

^*d*^Starting from 4.5 mmol EDA, **4** was obtained with 99% GC yield and 99% isolated yield. See ESI for details.

It is worth mentioning that the best catalysts described so far for the transformations considered usually bear electron-withdrawing groups on the ligand, making the metal electronically poor.^1^ Since such groups are not present in the structure of the PS–TTM in the catalyst employed herein the results obtained with this catalyst can be considered as quite good. Even more interesting are the catalyst recycling and reuse results. Yields remained essentially constant over the five reaction cycles with the most reactive substrates [tetrahydrofuran (**2**), ethanol (**3**), aniline (**4**), benzene (**5**), 1-phenyl-1-propyne (**6**)], and showed a slight decrease with the less reactive cyclohexane (**1**) in the C–H bond insertion reaction (see ESI† for detailed experimental protocols). To the best of our knowledge, the results shown in Table 1 represent the first example of Büchner reaction performed under heterogeneous conditions. In addition, the yields and selectivities recorded for the other studied substrates are, on average, better than those previously reported with heterogenized catalytic species involving the more expensive rhodium metal.^4c–e,6^

As a further assessment of the potential of the [(PS–TTM)Cu(NCMe)][PF_6_] catalyst, we decided to explore the possibility of using a single catalyst sample to carry out in a sequential manner the different transformations shown in Scheme 4. To test this possibility, a series of twelve consecutive experiments was performed with a 100 mg (0.039 mmol) sample of the catalyst, involving the carbene transfer from EDA (0.75 mmol) to each of the six substrates (each experiment was performed in duplicate). The reactions were performed in a Schlenk flask under an inert atmosphere, and the results are summarized in Table 2. Interestingly, no decrease in activity could be detected over the whole series of experiments, and compounds **1–6** were obtained in yields comparable to those recorded in the recycling study (Table 1). In addition, no cross-contamination arising from reactants or products from the previous reaction could be detected by GC after washing the catalyst with dichloromethane (2 × 5 mL) between cycles. This means, in practice, that a simple laboratory flask containing a small amount of the [(PS–TTM)Cu(NCMe)][PF_6_] catalyst performs as a general carbene transfer reactor, and can be used when required for different substrates and reaction types falling into this general class. Thanks to the high catalytic activity of the immobilized cationic Cu species, turnover numbers (TONs) up to 38 can be achieved in short reaction times. We are not aware of any report on a heterogeneous catalyst with such versatility for the functionalization of organic compounds by carbene transfer from diazo reagents.

**Table 2:** Consecutive use of the heterogenized catalyst for different substrates and reactions under batch conditions^*a*^

Cycle #	Substrate	Conversion^*b*^ [%]	Product	Yield^*b*^ [%]
1	Cyclohexane	98	**1**	73
2	Cyclohexane	97	**1**	65
3	Benzene	>99	**5**	72
4	Benzene	>99	**5**	62
5	1-Phenyl-1-propyne	98	**6**	85
6	1-Phenyl-1-propyne	99	**6**	92
7	Aniline	99	**4**	99
8	Aniline	97	**4**	97
9	Ethanol	99	**3**	98
10	Ethanol	98	**3**	98
11	Tetrahydrofuran	97	**2**	90
12	Tetrahydrofuran	99	**2**	94

^*a*^All reactions were performed with 5.2 mol% of catalyst loading.

^*b*^Yields were determined by GC and NMR. See ESI for details.

### Use of the [PS–TTM–Cu(NCMe)][PF_6_] catalyst under continuous flow conditions

**Fig. 1:**
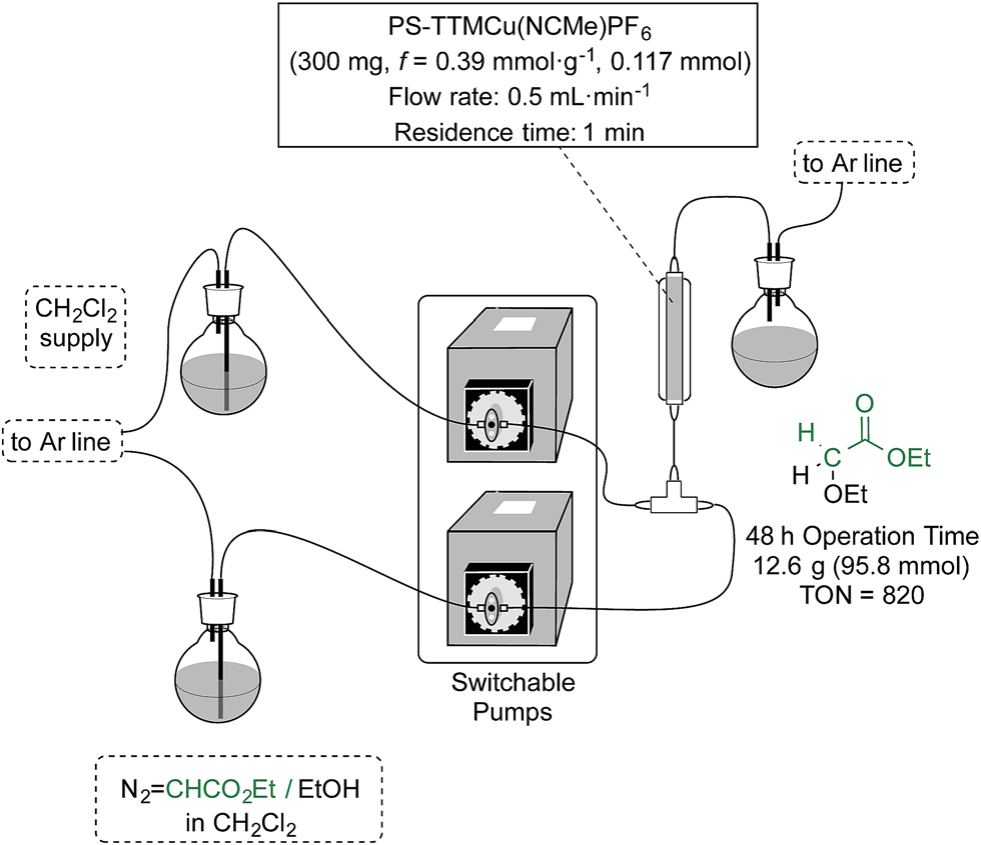
The experimental setup for continuous flow operation.

Upon obtaining successful results in the batch experiments, with a very stable and consistent performance, we moved to explore the catalytic system under continuous flow conditions. In fact, the additional effort required for the implementation of a flow process only finds adequate compensation when the TOF exhibited by the catalyst under the operation conditions remains constant over an extended period of time, so that the TON achieved with a given sample of catalyst scales linearly with the operation time.^15^ The experimental setup used for this study (Fig. 1) consisted of a vertically mounted and fritted low-pressure Omnifit column loaded with the [(PS–TTM)Cu(NCMe)][PF_6_] catalyst (see ESI† for details). A pump was used to feed the catalytic carbene transfer reactor with a dichloromethane solution of EDA and the substrate (no reaction takes place in the absence of catalyst) under an inert atmosphere. A second pump was used to wash the system with dichloromethane when required (*i.e.*, when the reactor has to be used with a different substrate).

**Fig. 2:**
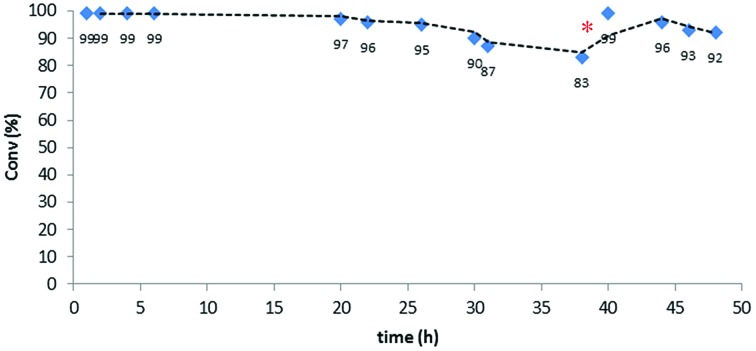
Continuous flow ethanol insertion to ethyl diazoacetate. Red dot indicates washing with dichloromethane.

The initial optimization of the reaction conditions was performed for the reaction of ethyl diazoacetate with ethanol at room temperature. The preliminary flow tests dictated some modifications with respect to the conditions optimized for batch processing. Since the swelling capacity of Merrifield resins is significantly reduced in ethanol, dilution of this reactant with dichloromethane was required. This efficiently prevented any contraction in the microporous structure of the polymer and facilitated optimal contact of the reactants with the catalytic sites. Gratifyingly, under optimized reaction conditions involving a flow rate as high as 500 μL min^–1^ (equivalent to 1 min residence time) the formation of the final product was achieved in very high yields. Thus, the reaction was performed using only 0.117 mmol of the [(PS–TTM)Cu(NCMe)][PF_6_] catalyst (300 mg; *ƒ* = 0.39 mmol g^–1^) and a solution of ethyl diazoacetate (11.2 mL, 106 mmol) and distilled ethanol (31 mL, 531 mmol) in deoxygenated dichloromethane (1400 mL). To our delight, even after 48 h both conversion and chemoselectivity remained above 90% (Fig. 2). After 38 h of operation, a slight decrease in conversion was observed, most probably due to contraction of the polymer matrix related to the pressure generated inside the column by the pumping process. To solve this issue, dichloromethane was pumped for 30 min at a flow rate of 500 μL min^–1^. This caused a visible re-swelling of the polymer matrix and increased the catalytic activity, so that full conversion was again reached. After this reactivation, the system was operational for at least ten more hours without any significant deterioration in conversion.

This very stable continuous flow process led, after 48 hours operation, to the isolation of 12.6 g (95.8 mmol) of EtOCH_2_CO_2_Et by simple evaporation of the volatiles in the effluent. This corresponds to a TON of 820 and to a productivity of 17.1 mmol_product_ mmol_Cu_^–1^ h^–1^.

Under these rather demanding conditions, the system was tested for Cu leaching. Very interestingly, quantitative analysis of the Cu content of samples of the effluent collected after an initial stabilization period indicated Cu contents between 0.8 and 1.6 ppm, thus confirming the high stability of [(PS–TTM)Cu(NCMe)][PF_6_] under operational conditions.

**Fig. 3:**
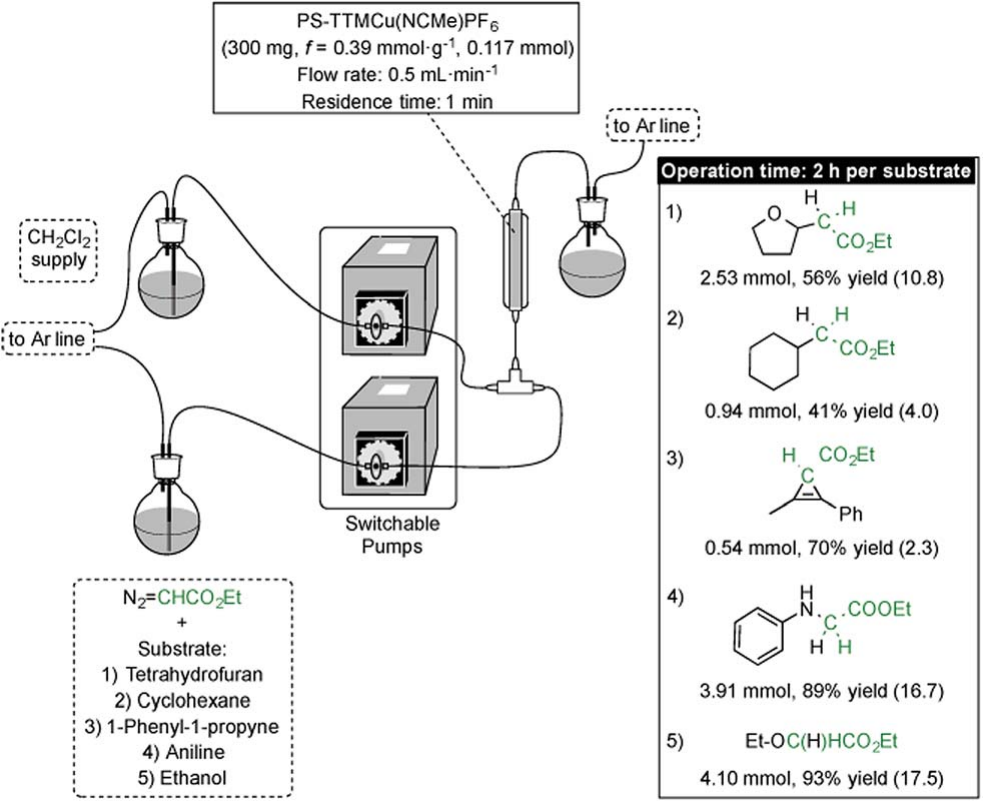
Sequential production in flow of a family of compounds resulting from different carbene transfer reactions. Productivities in mmol_product_ mmol_Cu_^–1^ h^–1^ are shown in parentheses.

In addition to their inherent advantages, such as increased safety, straightforward scale-up and highly simplified work-up, continuous flow processes based on supported catalysts offer the additional advantage of allowing the sequential preparation of small libraries of compounds.^16^ In the present instance, such a sequential process would not only be a proof of concept for the robustness and high activity of the catalyst, but it would also enable concise access to various carbene insertion products by sequential synthesis in a flow device. Given the high productivities showcased by the [(PS–TTM)Cu(NCMe)][PF_6_] catalyst either in batch or in a simple flow process (see above), we decided to assess the feasibility of this concept for the different types of carbene transfer reactions from EDA considered in this study. Hence, five different substrates involving four different types of carbene transfer (O–H insertion, N–H insertion, C–H insertion and cyclopropenation) were reacted with EDA in flow in the presence of the immobilized cationic Cu catalyst in a sequential manner. Each substrate/EDA combination was circulated through the packed-bed reactor for 2 h (flow rate = 500 μL min^–1^), with the column being rinsed by circulation of dichloromethane between two consecutive substrates (Fig. 3; see ESI† for details). Productivities ranging from 2.3 (cyclopropenation) to 17.5 mmol_product_ mmol_Cu_^–1^ h^–1^ (O–H insertion) were recorded. Except for the cyclopropenation case, productivities in flow are significantly higher (up to four times) than those recorded for the same reactions in batch (see ESI†). From a practical perspective, the robustness of the resin is further verified by the fact that the same 300 mg sample of [(PS–TTM)Cu(NCMe)][PF_6_] could be used without any deterioration of activity for the optimization of flow conditions for each substrate and for the synthesis in flow of the whole family of compounds resulting from carbene transfer. This remarkable stability would allow the use of a cartridge packed with [(PS–TTM)Cu(NCMe)][PF_6_] as a reusable, general carbene transfer catalyst that can be mounted/dismounted in a flow device whenever required.

## Conclusions

In summary, we have developed the first recyclable and highly efficient heterogenized copper complex for carbene transfer reactions to various types of substrates both in batch and, for the first time, in continuous flow. Remarkably, due to the high activity of the catalyst, residence times of 1 min can achieve complete conversion (0.117 mmol catalyst; single pass operation). The combined profile of the [(PS–TTM)Cu(NCMe)][PF_6_] catalyst with respect to activity, scope and chemical stability (under an inert atmosphere) makes this system one of the most promising developed to date for metal-mediated continuous flow processes. Studies aimed at its integration into synthetic sequences for the synthesis of complex molecules in flow are currently underway in our laboratories.
