# Supplementary material for: A fully recyclable heterogenized Cu catalyst for the general carbene transfer reaction in batch and flow
Source: Chem Sci. 2014 Nov 28;6(2):1510–5. doi: 10.1039/c4sc03277b (PMC5811104; doi:10.1039/c4sc03277b)
Supplement: Supplementary file 1 [file SC-006-C4SC03277B-s001.pdf]

Electronic Supplementary Material (ESI) for Chemical Science.
This journal is © The Royal Society of Chemistry 2014

Electronic Supporting Information

A Fully Recyclable Heterogenized Cu Catalyst for the General Carbene Transfer Reaction in Batch and Flow

Lourdes Maestre,[a] Erhan Ozkal,[b] Carles Ayats,[b] Álvaro Beltrán,[a] M. Mar Díaz-Requejo,[a,]* Pedro J. Pérez[a,]* and Miquel A. Pericàs[b,]*

[a]Laboratorio de Catálisis Homogénea, Unidad Asociada al CSIC, CIQSO-Centro de Investigación en Química Sostenible and Departamento de Química y Ciencia de Materiales, Universidad de Huelva , 21007 Huelva, Spain

[b]Institute of Chemical Research of Catalonia (ICIQ), Avda. Països Catalans 16, 43007, Tarragona, Catalonia, Spain.

[c]Departament de Química Orgànica, Universitat de Barcelona, 08028 Barcelona, Spain.

Table of contents

1. General Information S2

2. Synthesis of the polymer-supported copper complex PS-TTMCu(NCMe)PF_6. S2

3. Catalytic experiments under batch conditions S3

4. Description of the experimental setup for the continuous-flow process S5

5. Continuous-flow experiment with ethyl diazoacetate and ethanol S6

6. Continuous flow production of a family of compounds resulting from carbene transfer reactions S7

7. ^{1}H NMR and ^{13}C NMR data for products **1-6** S8

8. ^{1}H NMR and ^{13}C NMR spectra for products **1-6** S11

9. GC chromatograms for products **1-6** S17

1. General Information

Unless otherwise stated all the reactions were done under inert conditions (exclusion of air and moisture); under positive pressure of nitrogen (catalyst preparation and batch catalytic experiments) or argon (continuous flow experiments). Reactions were monitored by GC analyses performed on Agilent Technologies model 6890N gas chromatography instrument with a FID detector using 30 m x 0.25 mm HP-5 capillary column [He as carrier gas: 1.2 mL/min; initial temperature: 50 ºC (1.5 min); then heating at a rate of 10 ºC/min till 250 ºC (25 min)]. NMR spectra were recorded on a Bruker Advance 300 or 400 Ultrashield NMR spectrometers for ^{1}H NMR at 300 or 400 MHz and ^{13}C at 75 or 101 MHz. All samples were recorded in $CDCl_3$. Chemical shifts (δ) for protons are quoted in parts per million (ppm) downfield from tetramethylsilane and were referenced to residual proton resonances of the NMR solvent. Flash chromatography was carried out using 60 mesh silica gel and dry-packed columns. Commercial materials were used as received with following exceptions: all the solvents were used from a MBRAUN Solvent Purification System, and absolute ethanol was dried over Mg and further kept over activated 4 Å molecular sieves. Tetrakis(acetonitrile)copper(I) hexafluorophosphate, $[(CH_3CN)_4Cu]PF_6$, was purchased from Aldrich and was stored and used inside a glove box. Ethyl diazoacetate (EDA) was purchased from Aldrich and used as received. Tris(triazolyl)methane ligand[1] and polymer-supported ligand[2] were synthesized according to the previously published methods without any modifications. Elemental analyses and analysis of copper content (ICP-OES) were performed by Medac Ltd, Surrey (UK). The products of carbene transfer reactions were identified by comparison of their NMR spectra with those previously reported.[3]

2. Synthesis of the polymer-supported copper complex PS-TTMCu(NCMe)PF_6.

A mixture of PS-supported tris(triazolyl)methoxy ligand (300 mg, 1 equiv., f = 0.495 mmol·g^{-1}) and $[(CH_3CN)_4Cu]PF_6$ (65 mg, 0.176 mmol, 1.20 equiv.) was stirred in 20 mL of dry dichloromethane for 16 h under nitrogen. After filtration, the solid was washed with dry dichloromethane (2 x 20 mL) and petroleum ether (2 x 20 mL) and dried under vacuum. No changes in color were observed in the isolated solid from that of the starting material. In the event that some greenish color was observed, this would be indicative of partial oxidation to Cu(II). This problem can be avoided by working under inert atmosphere.

In the case of the catalyst for continuous flow, the procedure for the formation of the Cu(I) complex was carried out inside a glovebox, with the resin sample loaded in the flow column. The column was then taken outside from the glovebox, and was shaken at room temperature under positive pressure of argon for 16 h. The column was then connected to the flow instrument and was washed with dry dichloromethane (20 mL) with a flow rate of 500 µL/min prior to use.

---

[1] a) S. Özçubukçu, E. Ozkal, C. Jimeno, M.A. Pericàs, *Org. Lett.* 2009, 11, 4680–4683. b) E. Ozkal, P. Llanes, F. Bravo, A. Ferrali, M.A. Pericàs *Adv. Synth. Cat.* **2014**, *356*, 857-869.

[2] E. Ozkal, S. Özçubukçu, C. Jimeno, M.A. Pericàs *Catal. Sci. Technol.* **2012**, *2*, 195-200.

[3] a) M. J. Joung, J. H. Ahn, D. W. Lee, N. M. Yoon *J. Org. Chem.* **1998**, *63*, 2755-2757. b) P. Müller, C. Gränicher *Helv. Chim. Acta* **1993**, *76*, 521-534. c) M. E. Morilla, J. Molina, M. M. Díaz-Requejo, T. R. Belderraín, M. C. Nicasio, S. Trofimenko, P. J. Pérez *Organometallics* **2003**, *22*, 2914-2918. d) J. A. Flores, V. Badarinarayana, S. Singh, C. J. Lovely, H. V. R. Dias *Dalton. Trans.* **2009**, 7648-7652. e) L. K. Baumann, H. M. Mbuvi, G. Du, L. K. Woo *Organometallics* **2007**, *26*, 3995-4002. f) M. E. Morilla, M. M. Díaz-Requejo, T. R. Belderraín, M. C. Nicasio, S. Trofimenko, P. J. Pérez *Organometallics* **2004**, *23*, 293-295.

Elemental analysis of the resin PS-TTMCu(NCMe)PF_6:

N, 5.40%; C, 73.35%; H, 6.10%; Cu, 2.74%

The functionalization of the resin PS-TTMCu(NCMe)PF_6 (f = 0.39 mmol·g^{-1}) was calculated from the results of elemental analysis of nitrogen.[4] These results are consistent with those resulting from the analysis of Cu by ICP-OES.

3. Catalytic experiments under batch conditions

Carbene insertion into the O-H bond of ethanol

a) Small scale experiments: Dry and deoxygenated ethanol (5 mL) was added to a Schlenk flask containing PS-TTMCu(NCMe)PF_6 (100 mg, 0.039 mmol) under nitrogen atmosphere. EDA (0.75 mmol, 79 μL) was added in one portion and the mixture was stirred for 3 h at room temperature. The mixture was then filtered, the solid washed with CH_2Cl_2 (2 x 5mL), dried under vacuum and loaded again with solvent and reactants. The filtrate from the reaction mixture was analyzed by GC, identifying exclusively the product derived from ethanol functionalization[3] and some diethyl fumarate and maleate from the formal dimerization of the $CHCO_2Et$ units from EDA. The filtrate was taken to dryness, at 0º C to avoid evaporation of the product, and the residue was dissolved in $CDCl_3$. and analyzed by NMR.

Average productivity in batch after 5 cycles : 6.3 $mmol_{product}\cdot mmol_{Cu}^{-1}\cdot h^{-1}$

b) Preparative, large scale experiment: Dry and deoxygenated ethanol (30 mL) was added to a Schlenk flask containing freshly prepared PS-TTMCu(NCMe)PF_6 (600 mg, 0.234 mmol, recovered from the preparative experiment with aniline described below) under nitrogen atmosphere. EDA (4.5 mmol, 0.47 mL) was added in one portion and the mixture was stirred for 3 h at room temperature. The catalyst was separated by filtration, washed with CH_2Cl_2 (2 x 30 mL), dried and stored for further use. The filtrate from the reaction mixture was analyzed by GC, showing exclusively the peak corresponding to **3** (>99% conversion and >99% selectivity). Solvents were removed under reduced pressure (P = 150 mbar, 30 ºC) to a weight of 1.15 g. At this point, CH_2Cl_2 (7 mL) was added, and the solvents (dichloromethane and residual ethanol) were removed under reduced pressure (P = 400 mbar, 30 ºC) to give pure **3** (0.58 g, 4.41 mmol, 98% isolated yield).

[4] The degree of functionalization of a resin can be calculated from the results of elemental analysis with the formulas: $f_N = (0.714/n_N)\%N$, where n_N is the number of nitrogen atoms in the functional unit and %N is the percent of nitrogen provided by the elemental analysis. See: A. Bastero, D. Font and M. A. Pericàs *J. Org. Chem.* **2007**, *72*, 2460-2468. The corresponding formula from the results of Cu analysis is: $f_{Cu} = (0.157/n_{Cu})\%Cu$.

Carbene insertion into N-H bonds of aniline

a) Small scale experiments: Dry deoxygenated dichloromethane (3 mL) was added to a Schlenk flask containing PS-TTMCu(NCMe)PF_6 (100 mg, 0.039 mmol) under nitrogen atmosphere, followed by 3.75 mmol (0.34 mL) of freshly distilled aniline. A solution of 0.75 mmol (79 µL) of EDA in 10 mL of CH_2Cl_2 was slowly added over 6 h with the aid of a syringe pump. Workup was similar to that described above, removal of volatiles being performed at room temperature.

Average productivity in batch after 5 cycles : 3.0 $mmol_{product} \cdot mmol_{Cu}^{-1} \cdot h^{-1}$

b) Preparative, large scale experiment: Dry deoxygenated dichloromethane (15 mL) was added to a Schlenk flask containing freshly prepared PS-TTMCu(NCMe)PF_6 (600 mg, 0.234 mmol) under nitrogen atmosphere, followed by 22.5 mmol (2.0 mL) of freshly distilled aniline. A solution of 4.5 mmol (0.47 mL) of EDA in 30 mL of CH_2Cl_2 was slowly added over 6 h with the aid of a syringe pump. The catalyst was separated by filtration, washed with CH_2Cl_2 (2 x 30 mL), dried and used for the preparative experiment with ethanol (see above). The filtrate from the reaction mixture was analyzed by GC, showing exclusively the peak corresponding to **4** (>99% conversion and >99% selectivity). Dichloromethane was removed under reduced pressure (P = 400 mbar, 30 ºC), and excess aniline was evaporatively distilled using a rotary microdistillation equipment at 60-70 ºC/0.3 mbar. The residue in the flask was pure **4** (0.801 g, 4.47 mmol, 99% isolated yield).

Carbene insertion into C-H bonds of cyclohexane and tetrahydrofuran

Following the above procedure, cyclohexane (3 mL) was reacted with EDA (0.75 mmol, 79 µL) in the presence of the solid catalyst (100 mg, 0.039 mmol) under nitrogen atmosphere. EDA was slowly added for 18 h (dissolved in cyclohexane). Identical protocol and amounts of reactants were employed in the case of tetrahydrofuran. Workup was similar to that described above, removal of volatiles being performed at room temperature.

Average productivity for cyclohexane in batch after 5 cycles : 0.81 $mmol_{product} \cdot mmol_{Cu}^{-1} \cdot h^{-1}$

Average productivity for tetrahydrofuran in batch after 5 cycles : 0.95 $mmol_{product} \cdot mmol_{Cu}^{-1} \cdot h^{-1}$

Cyclopropenation of 1-phenyl-1-propyne .

3 mL of dry deoxygenated dichloromethane was added to a Schlenk containing the catalyst (100 mg, 0.039 mmol) under nitrogen atmosphere, followed by 3.75 mmol (0.42 mL) of 1-phenyl-1-propyne. A solution of 0.75 mmol (79 µL) of EDA in 10 mL of dichloromethane was slowly added over 6 hours. Workup was similar to that described above, removal of volatiles being performed at room temperature.

Average productivity in batch after 5 cycles : 2.9 $mmol_{product} \cdot mmol_{Cu}^{-1} \cdot h^{-1}$

<u>Büchner ring expansion of benzene</u>

3 mL of dry deoxygenated benzene was added to a Schlenk containing the catalyst (100 mg, 0.039 mmol) under nitrogen atmosphere. A solution of 0.75 mmol (79 µL) of EDA in 10 mL of benzene was slowly added for 6 h, before workup, that was similar to that described above, removal of volatiles being performed at room temperature.

Average productivity in batch after 5 cycles : 2.6 $mmol_{product} \cdot mmol_{Cu}^{-1} \cdot h^{-1}$

<u>Consecutive experiments with different substrates</u>

The above conditions were employed in a consecutive manner with the same initial loading of catalyst for an overall number of 12 experiments, two with each substrate (see Table 2 in the manuscript).

4. Description of the experimental setup for the continuous flow process

For the continuous flow experiments, the instrumental set-up shown in **Figure S1** was used. The packed bed reactor consisted of a vertical mounted and fritted low-pressure Omnifit glass chromatography column (10 mm bore size and up to maximal 70 mm of adjustable bed height) loaded with 300 mg of PS-TTMCu(NCMe)PF_6 (f = 0.39 $mmol \cdot g^{-1}$). The reactor inlet was connected to a three-way connector that allows switching between two channels, connected to an Asia120® flow chemistry system developed by Syrris. One channel was connected to a solution of ethyl diazoacetate (EDA) and distilled ethanol in deoxygenated dichloromethane under argon (no reaction occurs in the absence of catalyst) through Pump 1, and the other channel was connected to a flask containing deoxygenated dichloromethane to rinse the system through Pump 2. The reactor outlet was connected to a receiving flask, where the product was collected. Conversions of the product at any moment were determined by gas chromatography of periodically collected samples. At the end of the experiment the solvent was removed under reduced pressure to give the final product. During operation, all the system was kept under argon using either balloons (picture) or connections to an argon manifold.

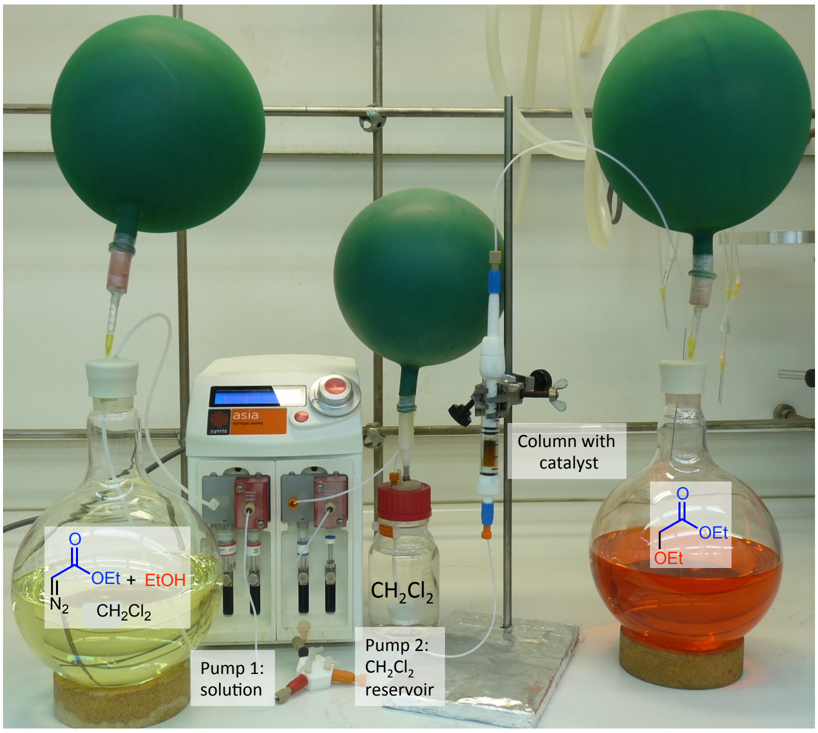

Figure S1. The experimental set-up for the continuous-flow experiment, *e.g.* with ethyl diazoacetate and ethanol.

5. Continuous flow experiment with ethyl diazoacetate and ethanol

The column was assembled to the Asia120® flow chemistry system developed by Syrris as shown in Figure S1. Deoxygenated dichloromethane was flushed by an Asian pump through the column at 500 μL·min^{-1} flow rate to swell the resin. After 30 min, the solvent channel was switched to the reagents and a solution of ethyl diazoacetate (11.2 mL, 106 mmol) and distilled ethanol (31 mL, 531 mmol) in deoxygenated dichloromethane (1400 mL) was pumped with a flow rate of 500 μL·min^{-1} through the system and the system was run for 48 h. Periodically collected samples were analysed by gas chromatography to determine conversion of the final product. Samples were collected and solvent was removed under reduced pressure to give pure product **3** (12.6 g, 95.83 mmol, 89 % yield) as a brownish liquid.

Productivity: 17.1 mmol$_{product}$ ·mmol$_{Cu}$$^{-1}$·h^{-1}; **TON**: 819 (referred to the product formed).

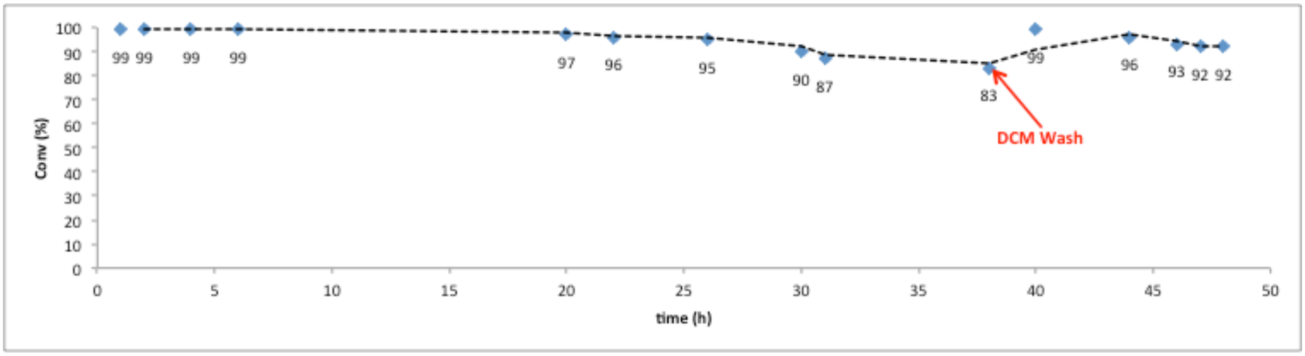

Figure S2. Conversion over time for continuous-flow reaction of ethanol and EDA.

6. Continuous flow production of a family of compounds resulting from carbene transfer reactions.

a) The column was filled with the catalyst as described above and assembled to an Asia120® flow chemistry system developed by Syrris (Figure S1). Deoxygenated dichloromethane was first flushed through the column at 500 $\mu L\cdot min^{-1}$ flow rate to ensure that no unligated Cu(I) is available. After 30 min, the solvent was changed to dry tetrahydrofuran, which was pumped through the column at 500 $\mu L\cdot min^{-1}$ flow rate. After 1 h, the solvent channel was switched to the reagents and a solution of EDA (0.56 mL, 5.3 mmol) in dry THF (70 mL) under argon was pumped with a flow rate of 500 $\mu L\cdot min^{-1}$. After a 10 min stabilization period, collection of the efluent was started and the process was kept running for 2 h. The collected samples were evaporated and analyzed by GC to determine conversion of the final product. The system was flushed with deoxygenated dichloromethane for 10 min at 500 $\mu L\cdot min^{-1}$ flow rate to clean the resin and the same procedure was repeated with the next substrate.

The solvent from the collected efluent was removed under reduced pressure (P = 200 mbar, 30 ºC) to give a yellow liquid which was submitted to flash column chromatography on silicagel (cyclohexane/ethyl acetate 95:5). The solvent was distilled off through a Vigreux column under reduced pressure (P = 200 mbar, 40 ºC) to give pure product **2** (0.40 g, 2.53 mmol, 56% yield).

Productivity: 10.8 $mmol_{product}\cdot mmol_{Cu}^{-1}\cdot h^{-1}$

b) Continuous flow reaction of EDA (0.28 mL, 2.65 mmol) and distilled and deoxygenated cyclohexane (35 mL, 0.32 mol) in deoxygenated DCM (35 mL).

The procedure described under **a)** was followed. Most of the solvent from the collected efluent was removed under reduced pressure (P = 400 mbar, 30 ºC) and residual solvent was distilled off through a Vigreux column under reduced pressure (P = 200 mbar, 40 ºC). The residue, a yellow liquid, was submitted to flash column chromatography on silicagel (cyclohexane 100%). The solvent of the collected fractions was distilled off through a Vigreux column under reduced pressure (P = 200 mbar, 40 ºC) to give pure product **1** (0.16 g, 0.94 mmol, 41% yield).

Productivity: 4.0 $mmol_{product}\cdot mmol_{Cu}^{-1}\cdot h^{-1}$

c) Continuous flow reaction of EDA (0.12 mL, 1.15 mmol) and 1-phenyl-1-propyne (1.3 mL, 10.4 mmol) in deoxygenated DCM (30 mL).[5]

The procedure described under **a)** was followed. The solvent from the collected efluent was removed under reduced pressure (P = 400 mbar, 30 ºC) to give a brown liquid, which was submitted to flash column chromatography on silicagel (cyclohexane/ethyl acetate 9:1). The solvent of the collected fractions was distilled off through a Vigreux column under reduced pressure (P = 200 mbar, 40 ºC) to give pure product **6** (0.11 g, 0.54 mmol, 70 % yield).

Productivity: 2.3 $mmol_{product}\cdot mmol_{Cu}^{-1}\cdot h^{-1}$

[5] In this experiment the flow rate was 175 μL/min.

d) Continuous flow reaction of EDA (0.56 mL, 5.3 mmol) and aniline (2.38 mL, 26.25 mmol) in deoxygenated DCM (70 mL).

The procedure described under **a)** was followed. The solvent from the collected efluent was removed under reduced pressure (P = 400 mbar, 30 ºC) to give a brown liquid which was submitted to bulb-to-bulb distillation at 60-70 ºC/0.3 mbar to remove traces of aniline. The material remaining in the flask was product **4** in pure form (0.70 g, 3.91 mmol, 89% yield).

Productivity: 16.7 $mmol_{product} \cdot mmol_{Cu}^{-1} \cdot h^{-1}$

e) Continuous flow reaction of EDA (0.56 mL, 5.3 mmol) and ethanol (1.54 mL, 26.4 mmol) in deoxygenated DCM (70 mL).

The procedure described under **a)** was followed. The solvent from the collected efluent was removed under reduced pressure (P = 400 mbar, 30 ºC) to give pure product **3** (0.54 g, 4.10 mmol, 93% yield).

Productivity: 17.5 $mmol_{product} \cdot mmol_{Cu}^{-1} \cdot h^{-1}$

7. ^{1}H NMR and ^{13}C NMR data for products 1-6.

Ethyl 2-cyclohexanylacetate, 1[3a]

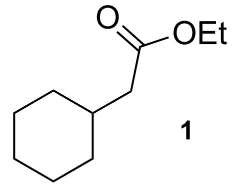

1

^{1}H-NMR (300 MHz, $CDCl_3$): δ = 0.93-1.02 (m, 2 H), 1.16-1.30 (m, 4 H), 1.25 (t, *J* = 6.9 Hz, 3 H), 1.63-1.75 (m, 5 H), 2.17 (d, *J* = 6.9 Hz, 2 H), 4.12 (q, *J* = 6.9 Hz, 2 H) ppm. ^{13}C-NMR (75 MHz, $CDCl_3$): δ = 14.3, 26.0, 26.2, 33.0, 34.9, 42.2, 60.1, 173.2 ppm.

Ethyl (tetrahydrofuran-2-yl)acetate, 2[3d]

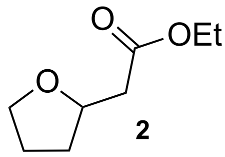

2

^{1}H-NMR (300 MHz, $CDCl_3$): δ = 1.26 (t, *J* = 7.2 Hz, 3 H), 1.49-1.59 (m, 1 H), 1.85-1.96 (m, 2 H), 2.03-2.14 (m, 1 H), 2.45 (dd, *J* = 15.0 and 6.3 Hz, 1 H), 2.59 (dd, *J* = 15.0 and 7.2 Hz, 1 H), 3.71-3.79 (m, 1 H), 3.84-3.92 (m, 1 H), 4.15 (q, *J* = 7.2 Hz, 2 H), 4.20-4.29 (m, 1H) ppm. ^{13}C-NMR (75 MHz, $CDCl_3$): δ = 14.2, 25.6, 31.2, 40.7, 60.5, 68.0, 75.3, 171.3 ppm.

Ethyl 2-ethoxyacetate, 3[3c]

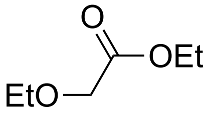

3

^{1}H-NMR (300 MHz, $CDCl_3$): δ = 1.24 (t, *J* = 6.9 Hz, 3 H), 1.28 (t, *J* = 7.2 Hz, 3 H), 3.58 (q, *J* = 6.9 Hz, 2 H), 4.05 (s, 2 H), 4.21 (q, *J* = 7.2 Hz, 2 H) ppm. ^{13}C-NMR (75 MHz, $CDCl_3$): δ = 14.1, 14.9, 60.7, 67.1, 68.0, 170.5 ppm.

Ethyl 2-(phenylamino)acetate, 4[3e]

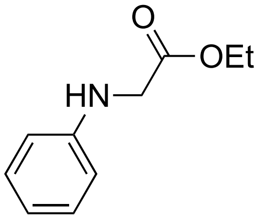

4

^{1}H-NMR (300 MHz, $CDCl_3$): δ = 1.30 (t, *J* = 6.9 Hz, 3 H), 3.9 (s, 2 H), 4.25 (q, *J* = 6.9 Hz, 2 H), 4.29 (br s, 1 H), 6.60-6.63 (m, 2 H), 6.73-6.78 (m, 1 H), 7.17-7.23 (m, 2 H) ppm. ^{13}C-NMR (75 MHz, $CDCl_3$): δ = 14.1, 45.8, 61.3, 112.9, 118.1, 129.3, 147.0, 171.1 ppm.

Ethyl cyclohepta-2,4,6-trienecarboxylate, 5[3f]

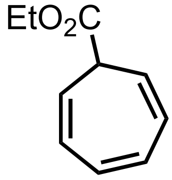

5

^{1}H-NMR (400 MHz, $CDCl_3$): δ = 1.31 (t, *J* = 7.2 Hz, 3 H), 2.53 (tt, *J* = 5.6 and 1.2 Hz, 1 H), 4.26 (q, *J* = 7.2 Hz, 2 H), 5.44 (dd, *J* = 9.6 and 5.6 Hz, 2 H), 6.24-6.28 (m, 2 H), 6.65-6.66 (m, 2 H) ppm. ^{13}C-NMR (101 MHz, $CDCl_3$): δ = 14.2, 44.1, 61.0, 117.3, 125.5, 130.9, 173.0 ppm.

Ethyl 2-methyl-3-phenylcycloprop-2-enecarboxylate, 6[3b]

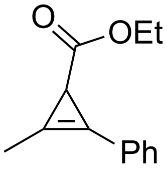

6

^{1}H-NMR (300 MHz, $CDCl_3$): δ = 1.25 (t, *J* = 7.2 Hz, 3 H), 2.33 (s, 3 H), 2.43 (s, 1 H), 4.15 (qd, *J* = 7.2 and 2.1 Hz, 2 H), 7.28-7.48 (m, 5 H) ppm. ^{13}C-NMR (75 MHz, $CDCl_3$): δ = 10.7, 14.4, 22.5, 60.1, 105.2, 106.3, 127.1, 128.5, 128.6, 129.2, 175.7 ppm.

8. ^{1}H NMR and ^{13}C NMR spectra for products 1-6.

Ethyl 2-cyclohexanylacetate, 1

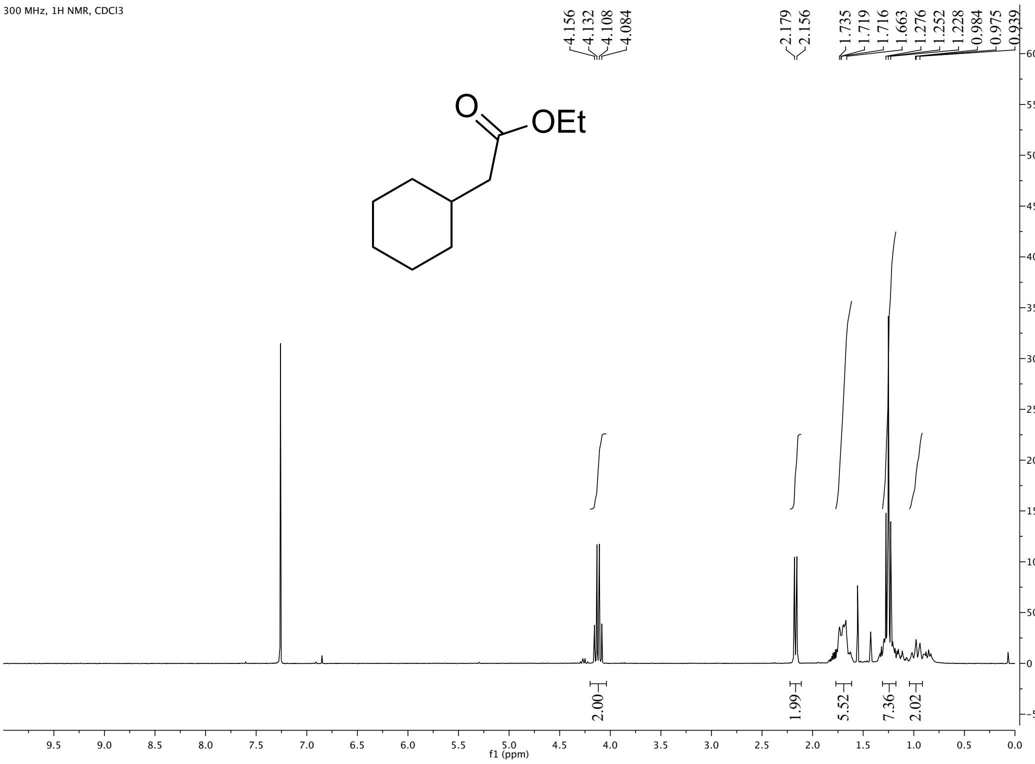

^{1}H NMR (300 MHz, CDCl$_3$)

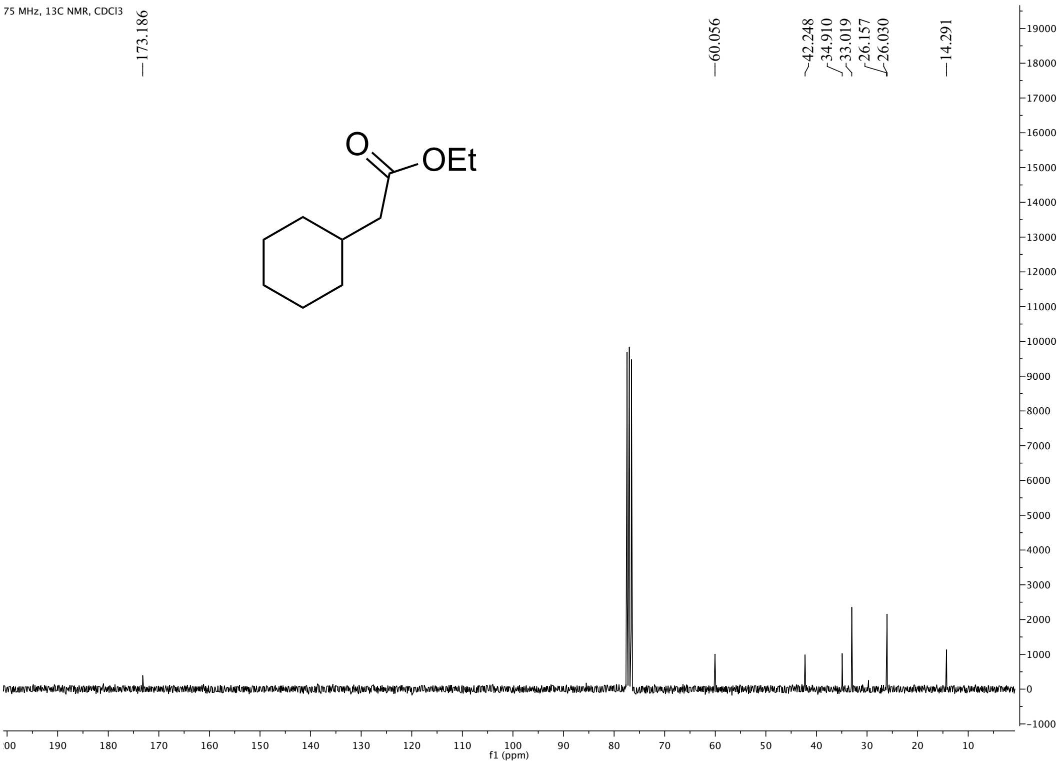

^{13}C NMR (75 MHz, CDCl$_3$)

Ethyl (tetrahydrofuran-2-yl)acetate, 2

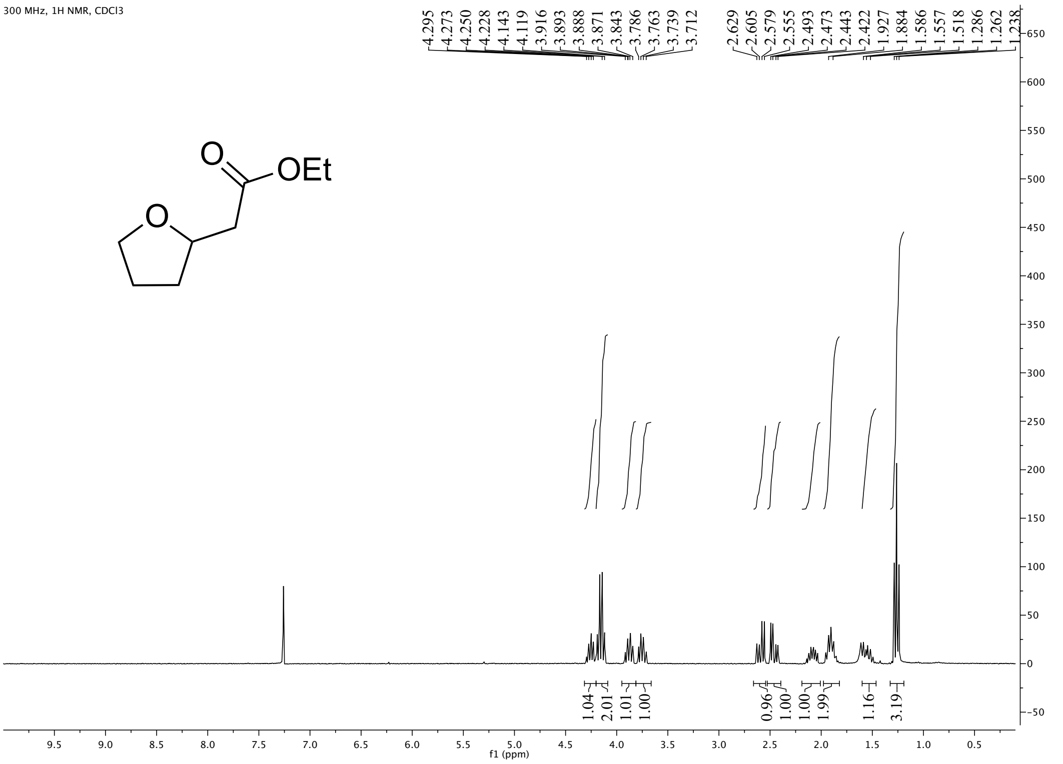

^{1}H NMR (300 MHz, $CDCl_3$)

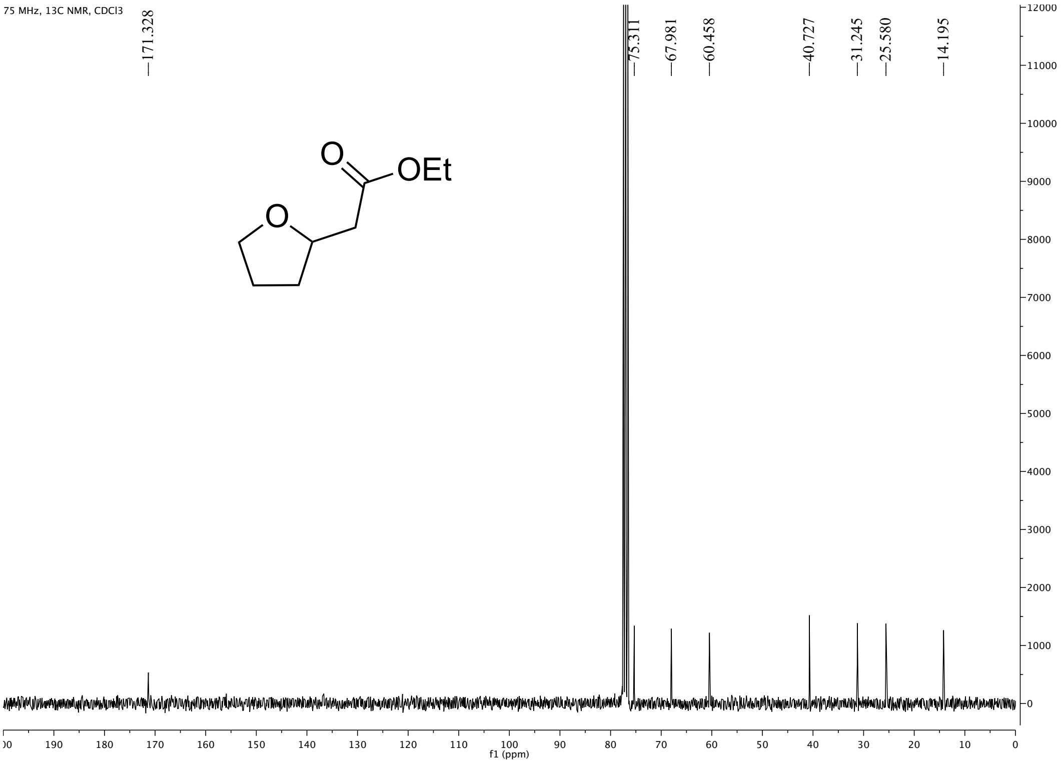

^{13}C NMR (75 MHz, $CDCl_3$)

Ethyl 2-ethoxyacetate, 3

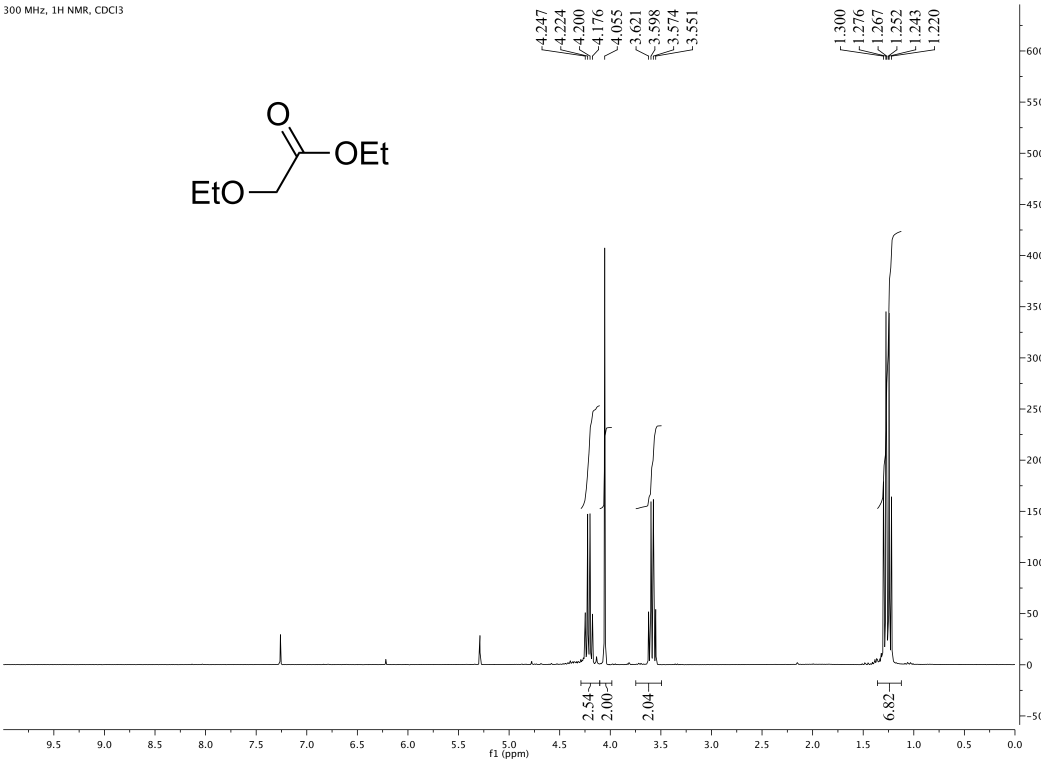

^{1}H NMR (300 MHz, $CDCl_3$)

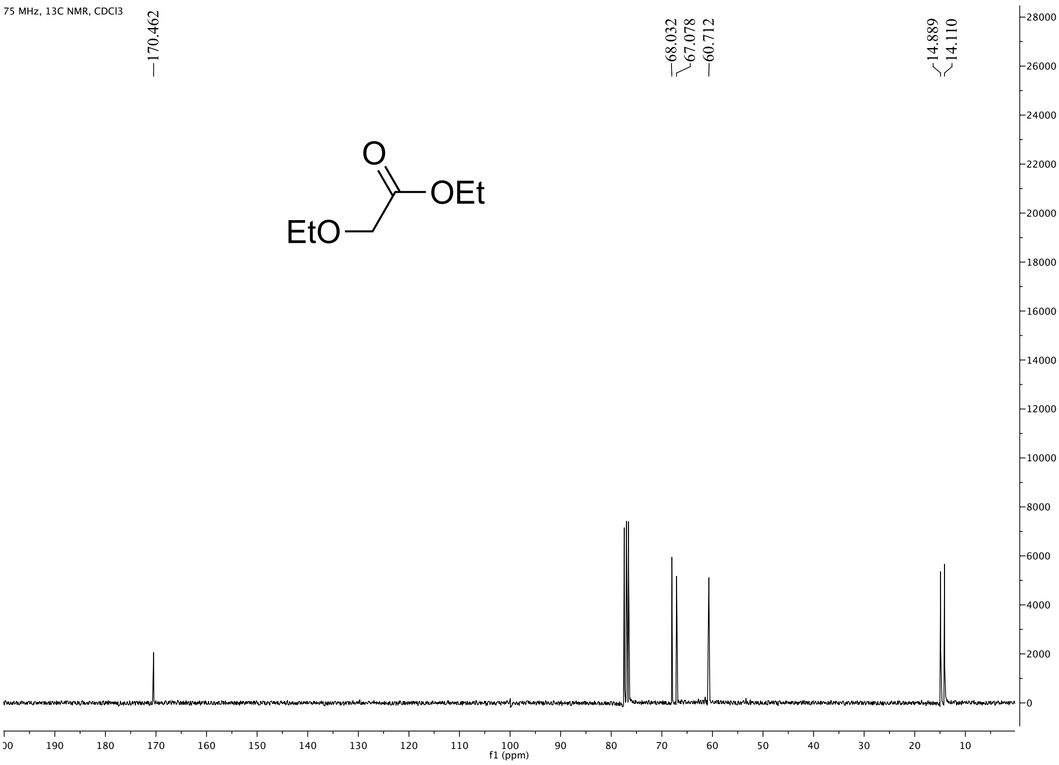

^{13}C NMR (75 MHz, $CDCl_3$)

Ethyl 2-(phenylamino)acetate, 4

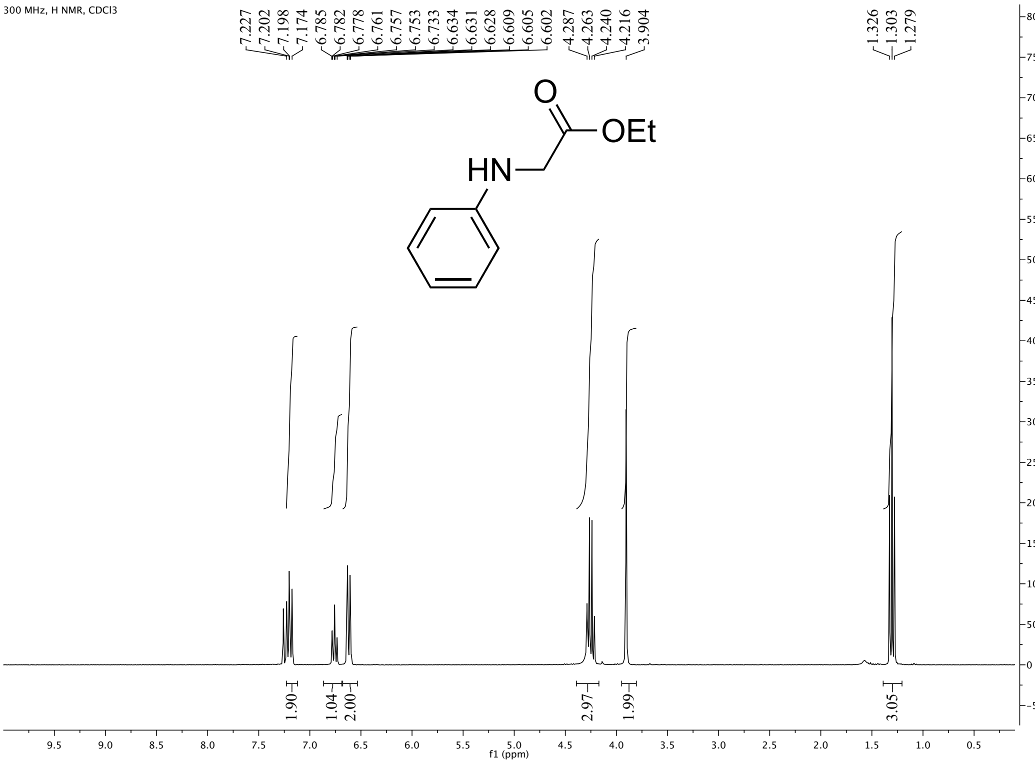

^1H NMR (300 MHz, $CDCl_3$)

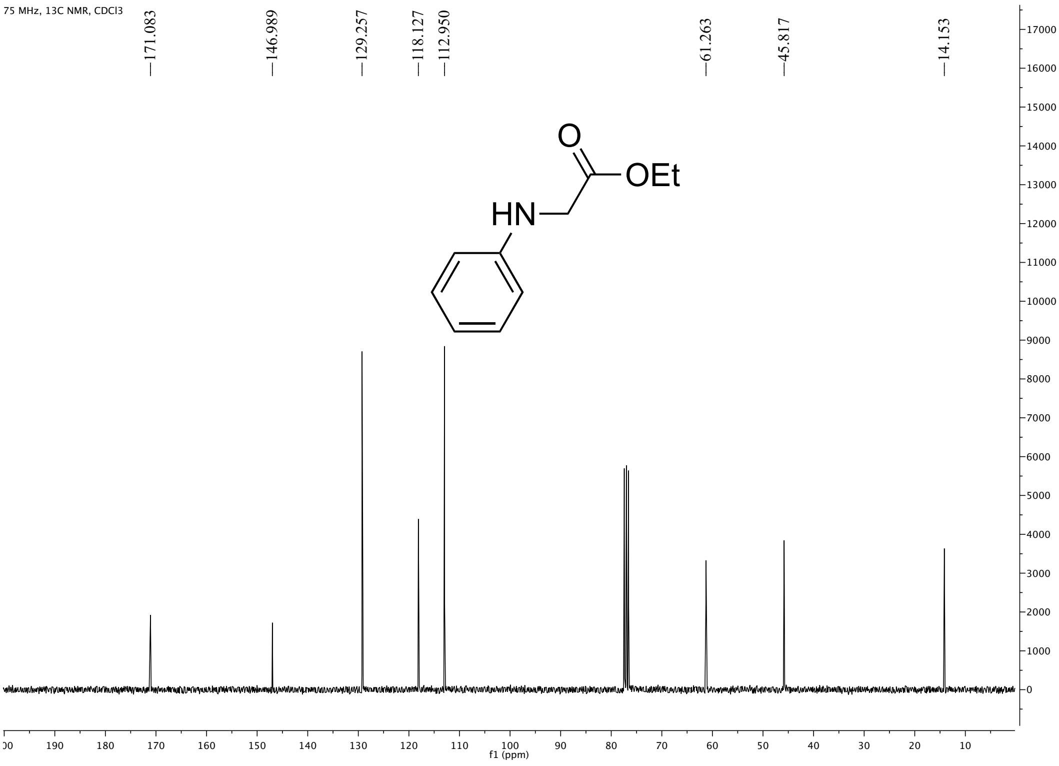

^{13}C NMR (75 MHz, $CDCl_3$)

Ethyl cyclohepta-2,4,6-trienecarboxylate, 5

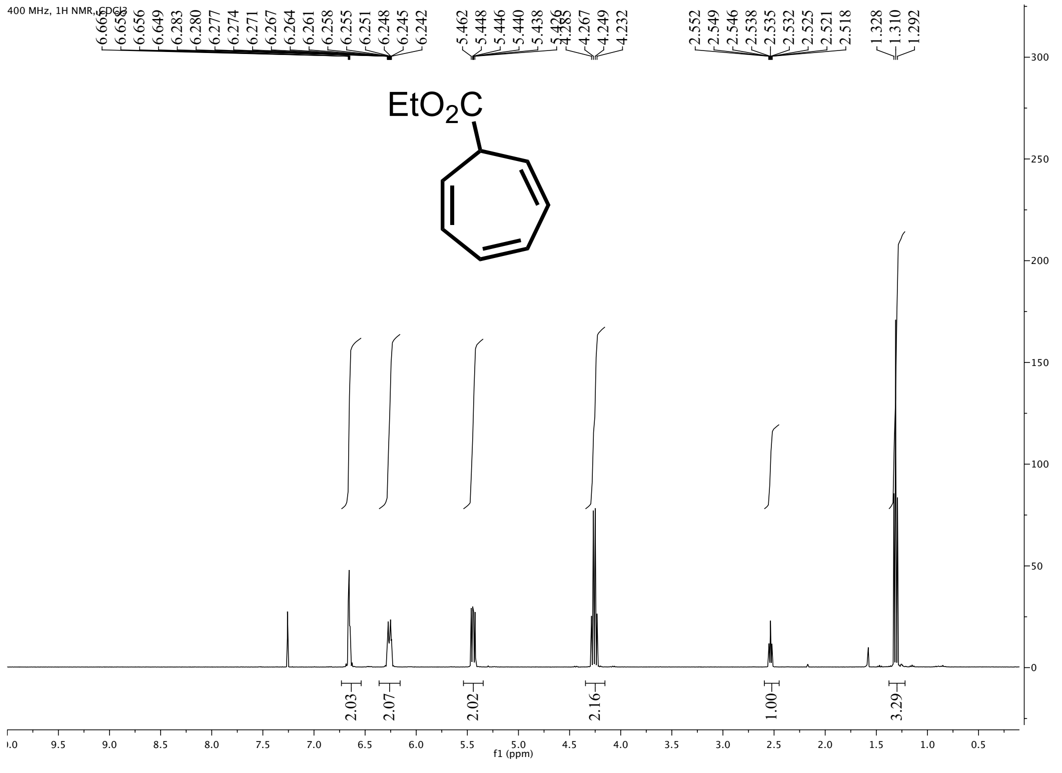

^{1}H NMR (400 MHz, $CDCl_3$)

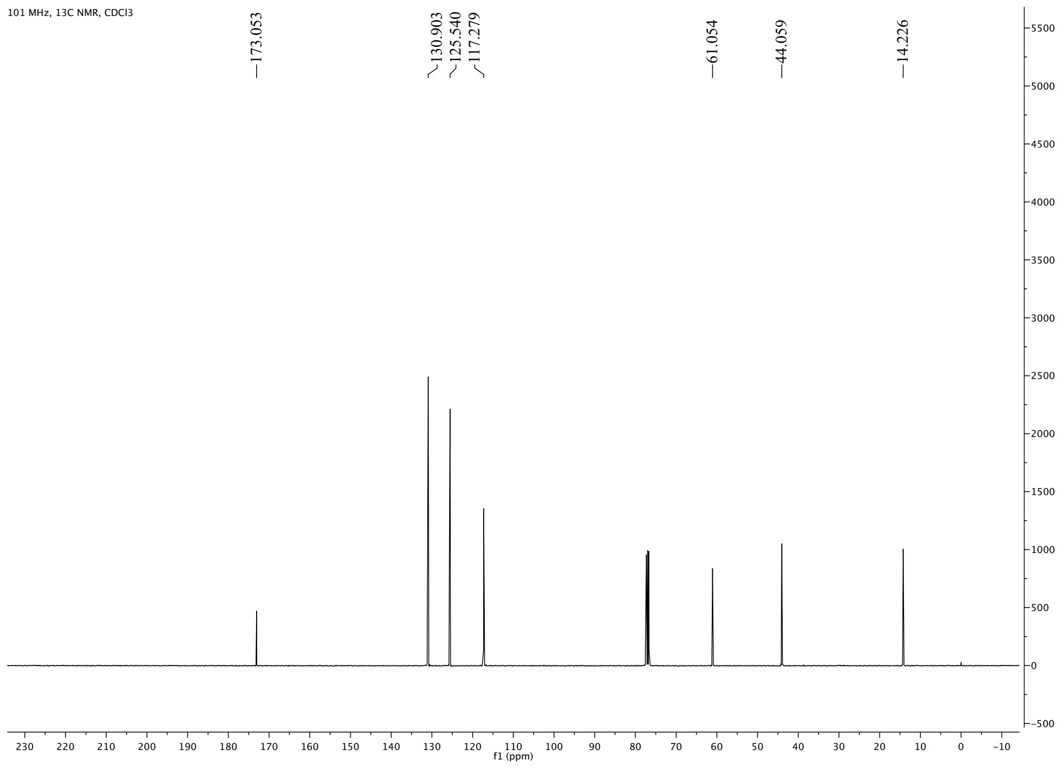

^{13}C NMR (101 MHz, $CDCl_3$)

Ethyl 2-methyl-3-phenylcycloprop-2-enecarboxylate, 6

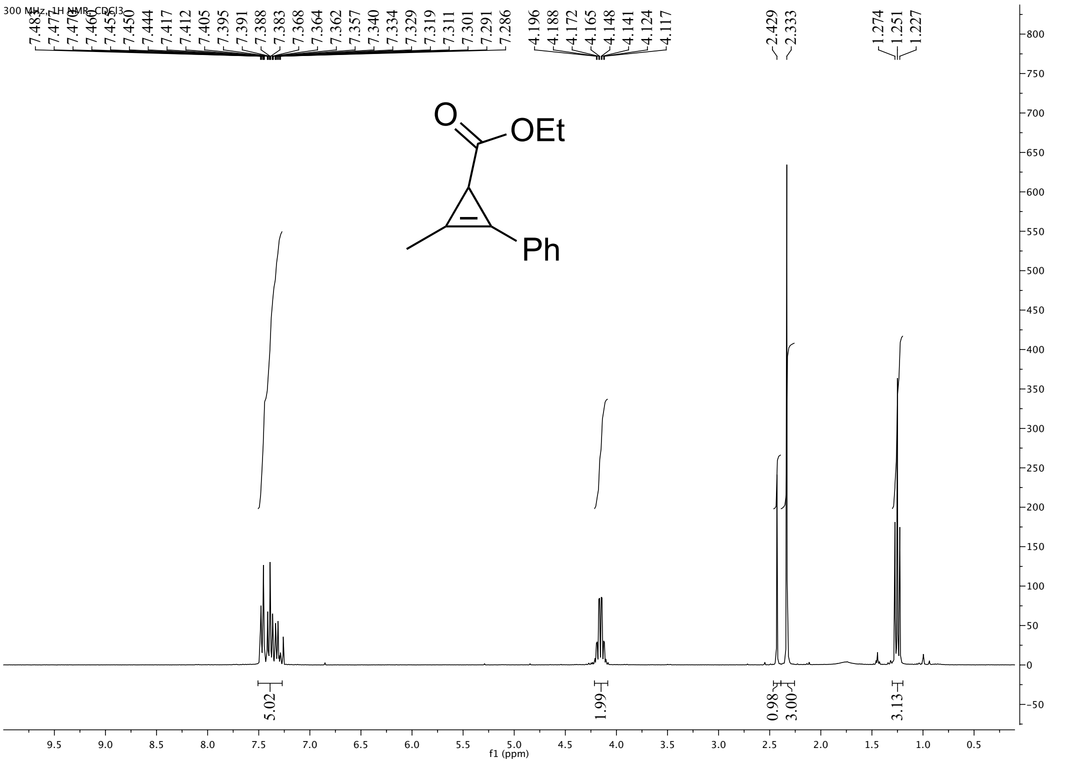

^{1}H NMR (300 MHz, $CDCl_3$)

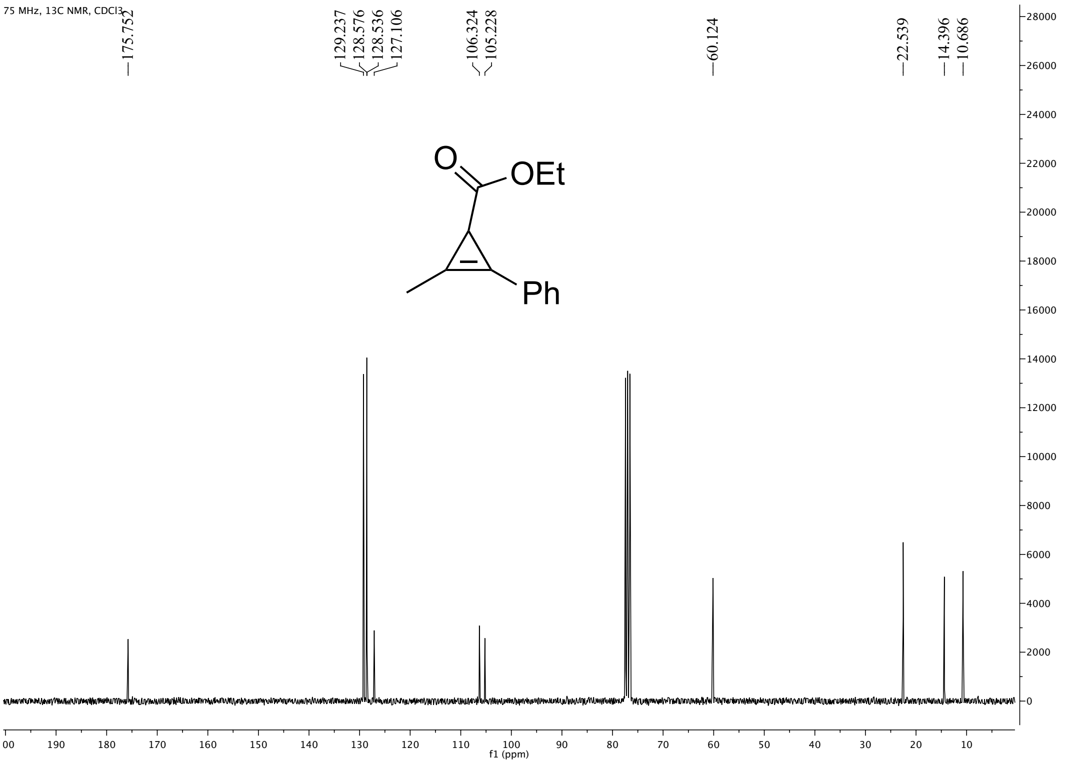

^{13}C NMR (75 MHz, $CDCl_3$)

9. GC chromatograms for products 1-6.

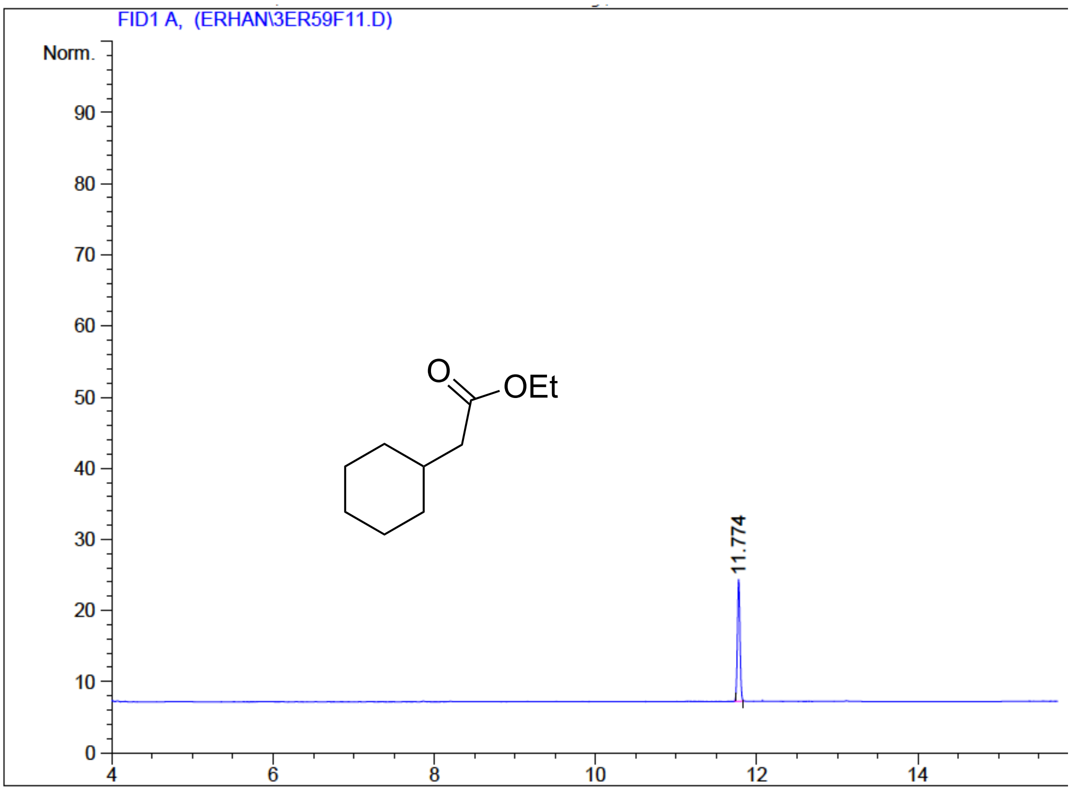

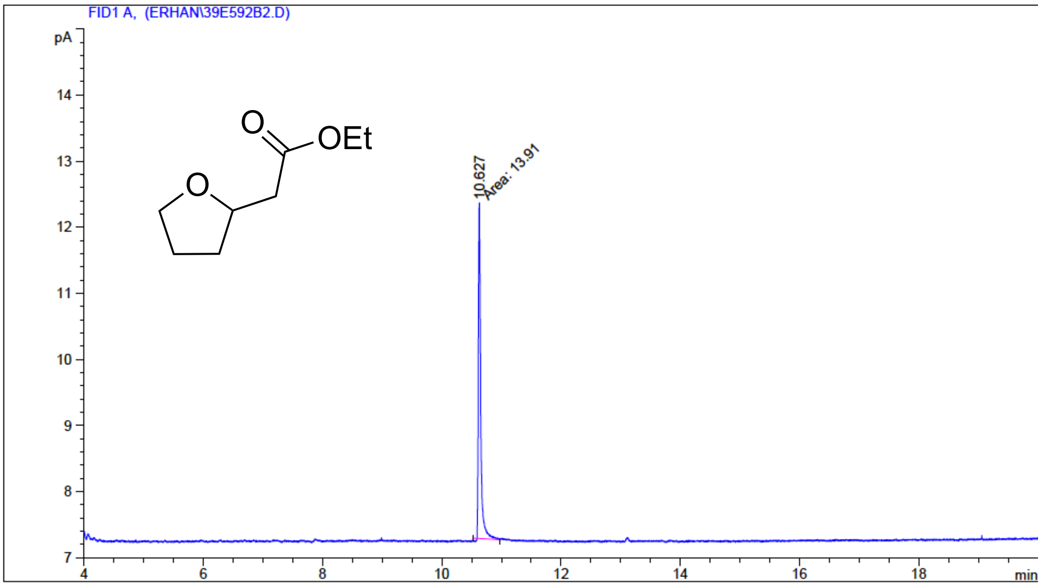

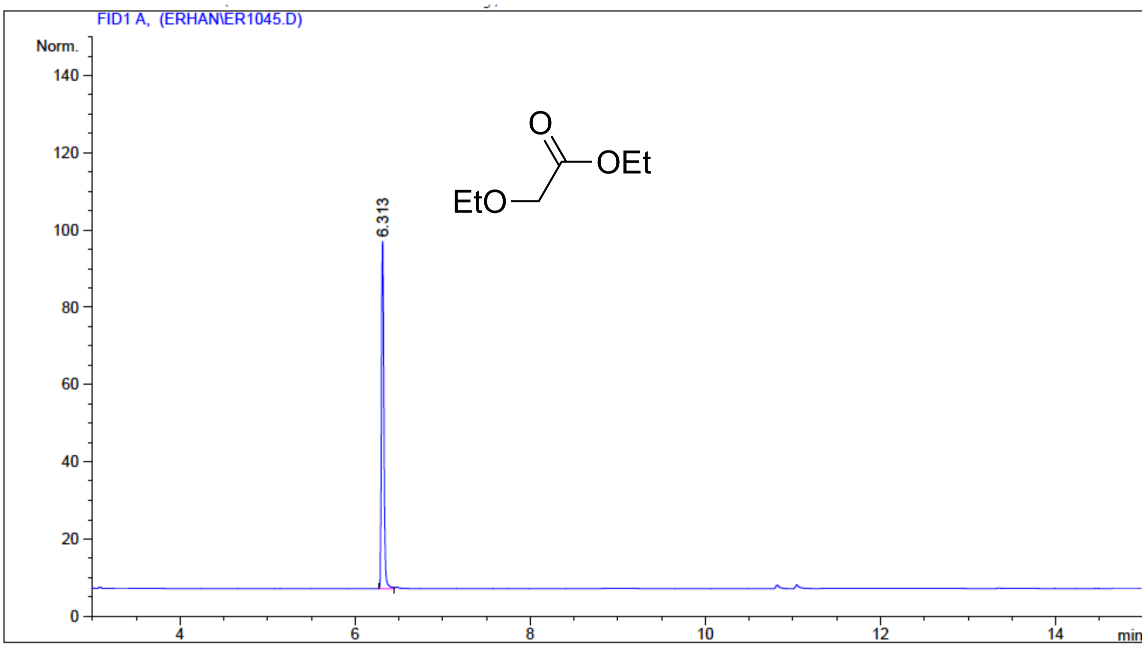

FID1 A, (ERHAN\ER1045.D)
Norm.
140
120
100
80
60
40
20
0
6.313
O
OEt
EtO
4
6
8
10
12
14
min

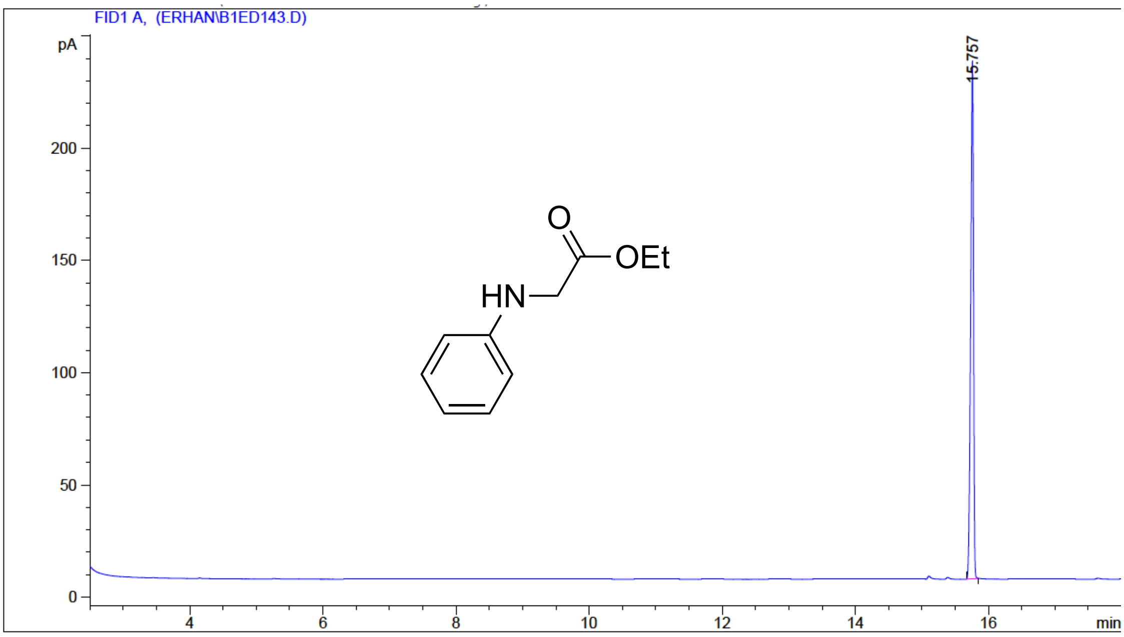

FID1 A, (ERHAN\B1ED143.D)
pA
200
150
100
50
0
15.757
O
OEt
HN
4
6
8
10
12
14
16
min

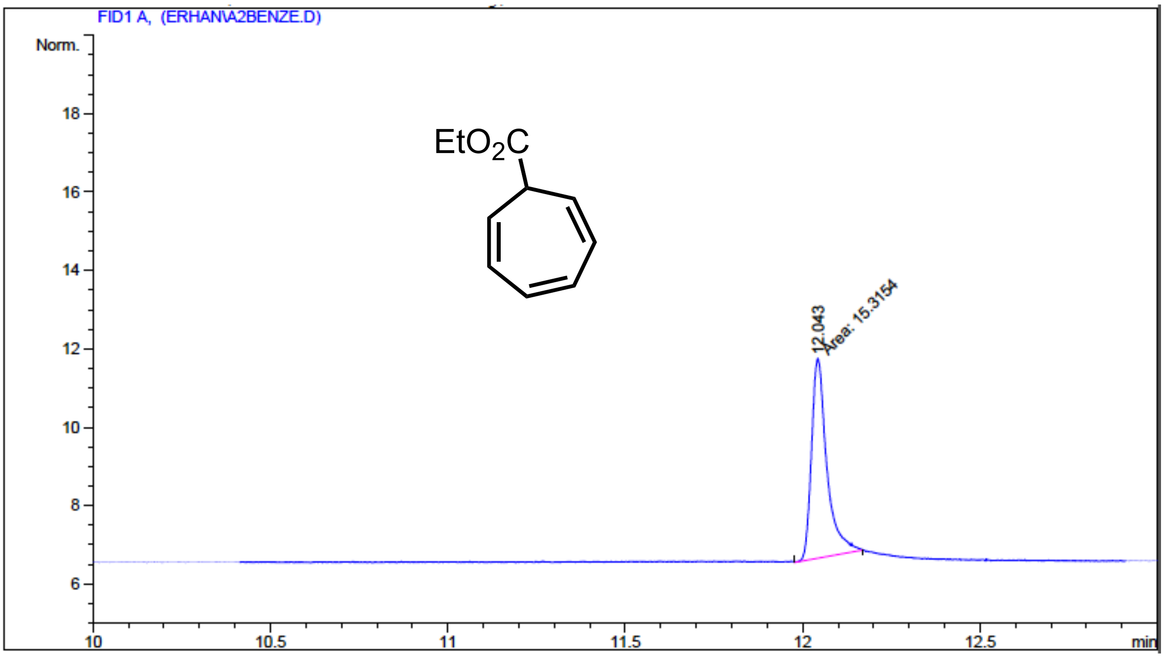

FID1 A, (ERHAN\A2BENZE.D)
Norm.
EtO2C
12.043
Area: 15.3154
min

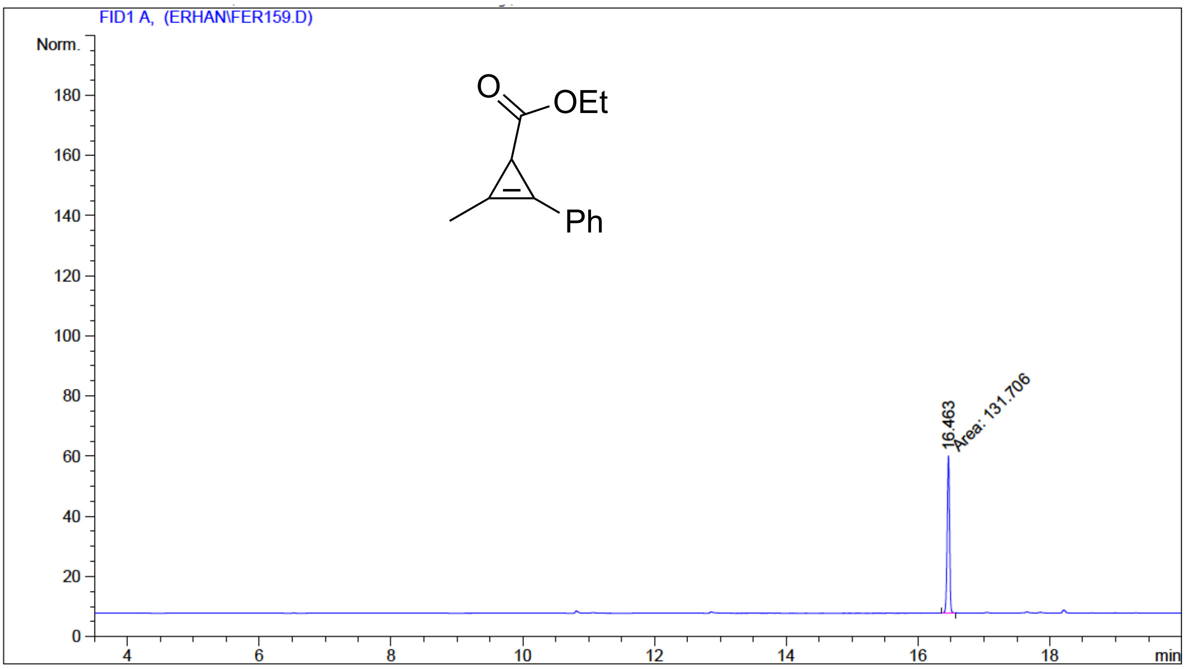

FID1 A, (ERHAN\FER159.D)
Norm.
O
OEt
Ph
16.463
Area: 131.706
min
